# Predictive analysis of seismic damage to buildings near-surface faults under the influence of multiple factors

**DOI:** 10.1371/journal.pone.0320930

**Published:** 2025-05-07

**Authors:** Jianyi Zhang, Zishan Bai, Jianke Ma, Shihang Qu, Jing Tian, Shuai Wang, Ran Zhang

**Affiliations:** 1 School of Geological Engineering, Institute of Disaster Prevention, Hebei, China; 2 Key Laboratory of Building Collapse Mechanism and Disaster Prevention, China Earthquake Administration, Hebei, China; 3 Hebei Key Laboratory of Earthquake Disaster Prevention and Risk Assessment, Hebei, China; 4 School of Civil Engineering, Institute of Disaster Prevention, Hebei, China; Islamic Azad University Mashhad Branch, IRAN, ISLAMIC REPUBLIC OF

## Abstract

The investigation and analysis of building damage is the most direct and effective method to reveal its mechanism, but the statistics and analysis of building damage sample data near surface faults are obviously insufficient. Therefore, based on the influencing factors of building damage near surface faults, this article predicts the structural damage caused by the surface rupture effect of active faults. Through on-site investigation of earthquake damage and literature review, data collection was conducted on the seismic damage characteristics of buildings near surface fault zones, and a database of building damage near strong earthquake surface fault zones was established. Multiple linear and binary logistic regression models were used to quantitatively analyze the seismic damage of buildings. The results indicate that magnitude, types of fault, hanging wall and footwall of faults, width of surface rupture zone, fault distance, and vertical displacement have a significant impact on the seismic damage index of buildings. Based on earthquake damage examples, both prediction models have significant performance.

## 1. Introduction

The surface rupture zone is a direct manifestation of the sudden rupture of rocks at the epicentre and their rapid propagation to the surface[1–3]. Research has shown that the structural damage and casualties of buildings (structures) on the surface rupture zone often far exceed those on both sides, and the degree of structural damage on the surface rupture zone is closely related to the parameters of fault rupture[4–6]. For example, the damage to buildings near the surface rupture zone on a reverse fault site is more severe than that of a normal fault [7]; The damage caused by the rupture of the dip-slip fault surface to the hanging wall buildings is greater than that to the footwall buildings [8,9].

In the standardization and related research, only general analysis has been provided on rupture zones and avoidance distances. According to UBC97 [10], four conditions must be met simultaneously: (1) the building is located in seismic fortification zone 4; (2) A fault is a seismic fault; (3) The fault has the ability to generate earthquakes of no less than M6.5 magnitude; (4) Only when the fault distance is less than 15 km, the near fault coefficient needs to be considered. However, UBC97 does not directly provide specific numerical values or calculation formulas for the near fault coefficient. In 1999, the California government revised the Alquist Priolo Fault Zoning Act [11], prohibiting the construction of buildings within 15 meters on each side of the fault trace; When the active fault is not perpendicular or the positioning is complex, a 300m fault avoidance zone (or 150m on each side of the trace) should be provided on a large active fault; Provide a 120-180m fault avoidance zone on a deterministic small active fault. In 2003, the Utah Department of Natural Resources [12] conducted a study on deterministic faults with a width of 150m in the footwall wall and 75m in the hanging wall; For concealed or approximately located faults, the width is 300m per plate. In 2003, the New Zealand Geological Atomic Energy Agency [13] proposed that the width of the potential area of fault surface rupture hazard was between 10m and 50m. Due to the uncertainty of positioning, an additional 20m buffer was added to the outside of the potential area, forming a recommended hazardous area with a width of 50m to 90m. In 2006, the European Technical Committee [14] pointed out in its summary report that both sides of the strike-slip fault avoided the fault trace by 30 meters, the hanging wall of the normal and reverse faults avoided the fault trace by 30 meters, and the footwall of the normal and reverse faults avoided the fault trace by (30 + 1.5H) m and (30 + 2H) m, respectively. The Chinese Code for Seismic Design of Buildings [15] specifies the minimum avoidance distance for Class A, B, and C buildings in the event of seismic rupture as follows: For Class A buildings, due to their high importance, stricter avoidance measures need to be taken when facing seismic fractures. However, the “Code for Seismic Design of Buildings” does not directly provide the specific minimum avoidance distance for Class A buildings in the event of seismic rupture, but instead requires “specialized research”. For Class B buildings, the minimum avoidance distance for seismic faults is 200 meters when the seismic fortification intensity is 8 degrees; When the seismic fortification intensity is 9 degrees, the minimum avoidance distance is increased to 400 meters. For Class C buildings, the minimum avoidance distance for seismic faults is 100 meters when the seismic fortification intensity is 8 degrees; When the seismic fortification intensity is 9 degrees, the minimum avoidance distance is 200 meters.

Although various countries have proposed specific values for avoidance distance, there is no sufficient and reasonable explanation or verification data[16]. Considering the rapid development of social urbanization in areas with frequent active faults and the relatively weak disaster prevention caused by land scarcity, the current evaluation method for strong earthquake surface rupture zones caused a significant waste of land, constrained urban planning and development, and is there still feasibility for reducing the avoidance distance of buildings. Furthermore, what would happen if the surface ruptures through buildings and structures (foundations)? Do different buildings (structures) have the same resistance to surface rupture zones? Can surface rupture traces really bypass building foundations? How to formulate corresponding reinforcement design strategies and measures for the anti-fracture problems of the foundation and superstructure of existing buildings (structures) within the avoidance range, which inevitably requires more refined research conclusions or quantitative results on the relationship between the fracture zone and the buildings (structures).

**Fig 1:**
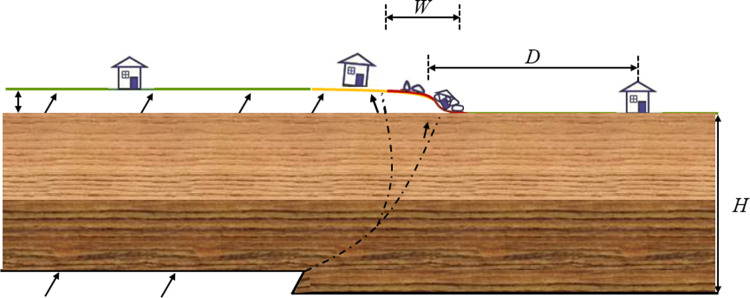
The impact of reverse fault surface rupture on buildings.

In fact, the degree of damage to buildings under the action of surface faults depends on various factors, as shown in Fig 1, including Types of fault(F), Width of surface rupture zone(W), Thickness of soil cover layer(H), Distance between buildings and surface rupture zone(D), Types of building structure, etc. These factors together constitute the direct influencing factors (input variables) that affect the degree of building damage, and the damage index of the building serves as the response value (output variable Y) under the influence of these factors.This can be expressed by the formula *f*_*(H, D, F, W….)*_*=Y*.

Therefore, how to avoid active faults and study fracture resistance in site selection for engineering construction is a concern in the field of geotechnical engineering and seismic design. The series of research works on the mechanism of soil rupture on active faults, passive avoidance, and active anti-fracture measures is currently a hot topic in engineering and academic circles [17–21]. The current research on the impact of surface rupture and seismic damage can be divided into two stages. In the first stage, researchers mainly focused on free sites (excluding building structures) and conducted an in-depth analysis of the degree of surface rupture caused by surface faults by integrating data on surface rupture damage, conducting model experiments, and using numerical simulations. In the second stage, surface rupture and the resulting structural damage gradually became the research core.

In the first stage, the research on the surface rupture of fault sites mainly relies on several methods such as seismic damage statistics, model experiments, and numerical simulations, and the theories of model experiments and numerical simulations are both based on statistics [22]. Zhao Lei et al. [23] conducted a comprehensive analysis of previous research results, summarized the fracture laws of overlying soil layers, and discussed the problems and development trends in research. They pointed out that the movement form, displacement, direction, thickness, and physical properties of the overlying soil layer of the fault are the main factors affecting soil layer rupture. Guo Tingting et al. [24] used the finite element numerical simulation method to analyze the characteristics of surface deformation and rupture of overlying soil layers and their influencing factors. Their results indicate that there is a significant relationship between fault dip angle, fault displacement, and overlying soil thickness and surface rupture. Lade et al. [25] determined through sandbox experiments that the location of surface fractures is related to the position of fault bedrock, soil layer thickness, fault misalignment angle, and soil expansion angle. Li Lianhui [26] combined the Okada method and numerical calculation method to analyze the close relationship between surface fault displacement and overlying soil thickness, fault displacement, and fault dip angle. Bo Jingshan et al. [27] proposed a feasible method for predicting the probability of surface rupture during strong earthquakes based on logistic regression and proposed evaluation factors and grading methods. Based on statistical regression methods, He Yalin [28] established an empirical regression relationship between the surface displacement response index of fault displacement and various influencing parameters and proposed a calculation method for the maximum surface displacement, maximum relative surface displacement, and surface rupture zone width of overlying clay and overlying sand on bedrock under different fault displacements.

In the second stage of research, many domestic scholars have started to quantitatively analyze the relationship between surface rupture and adjacent engineering structural damage, and have conducted preliminary analysis. In his research on the Jiji earthquake in Taiwan, Lin Fengtian[29] analyzed the spatial distribution pattern between the proportion of completely damaged buildings and the distance from the Chelongpu fault, revealing that the collapse rate of buildings within 100 meters of the surface rupture zone significantly increases with decreasing distance, and the degree of damage to buildings on the hanging wall of the fault is generally higher than that on the footwall. Zhao Jisheng et al. [30] conducted a detailed investigation on the extent of damage to 1699 buildings on the surface rupture zone in response to the Wenchuan earthquake. The research results show that the collapse rate of buildings within a distance of 150m from the surface rupture trace gradually decreases with increasing distance, while the integrity rate of buildings within a distance of 250m from the surface rupture trace gradually increases. Guo Tingting [4] further analyzed the correlation between the width of the ground rupture zone and the characteristics of building damage during the Wenchuan earthquake. By evaluating the damage level of buildings, it was found that the degree of damage to buildings of the same structural type gradually decreases with increasing distance from the fault. Zhang Jianyi [31] conducted an in-depth investigation of building damage near the Yushu earthquake fault and used linear regression to fit the expression I = 0.7969-0.00231D for the attenuation of the building damage index with the distance of the surface rupture zone. A. Alothman et al. [32,33] analyzed the influence of building height and seismic characteristics on the seismic performance of reinforced concrete buildings and concluded that as the number of floors increases, their vulnerability to earthquakes also increases. At the same time, different types of ground movements have a significant impact on the seismic performance of RC buildings, especially near fault earthquakes, which often cause greater damage to high-rise RC buildings due to their unique velocity and displacement pulse characteristics. Meng YaTian et al. [34] took the Sichuan region as an example and optimized the BP neural network through an improved genetic algorithm to establish an evaluation model that can comprehensively consider the impact of different seismic damage factors on the damage level of individual buildings, providing a new idea and method for regional seismic damage assessment. Reza M.S. et al. [35] used fuzzy logic methods to evaluate the damage to buildings at earthquake sites. The input parameters of this model include building height and age, soil shear wave velocity, plane equivalent moment of inertia, fault distance, earthquake acceleration, number of residents, and street width, among others, while the output parameters are the damage level of the building. Rahmani Qaranqayeh M. et al. [36] proposed an innovative two-stage sampling method, combined with the Kriging regression model, providing a new approach and method for predicting building damage after earthquakes. This method not only significantly improves the accuracy and reliability of predictions, but also provides strong decision support for earthquake disaster management.

In summary, the most convincing and reliable data in these research works is the analysis of seismic damage characteristics of sites and buildings near the surface rupture zone of strong earthquakes. However, the number of previous studies is limited, resulting in mostly qualitative conclusions and a lack of quantitative indicators. The results are difficult to guide engineering practice and provide reasonable avoidance distances; It is more difficult to provide the design of anti-fracture measures for the building itself and its covering area within a certain avoidance distance.

Although previous studies have achieved certain results, there is still a significant gap in the comprehensive connection and statistical analysis of the seismic damage characteristics of buildings significantly affected by surface rupture in different major earthquakes. In short, there is currently no systematic integration and extensive statistical analysis of such seismic damage data, and no analysis of seismic damage characteristics.

The purpose of this article is to collect seismic damage data for the “surface rupture zone-overburden layers-foundation-super structures” system through on-site investigation and literature review methods, establish a seismic damage database for buildings near the strong earthquake surface rupture zone, adopt appropriate mathematical models for seismic damage characteristic analysis, and reasonably and safely predict the seismic damage index of near fault buildings, in order to provide an important scientific basis for the revision and improvement of seismic design specifications and achieve the organic combination of scientific research innovation and engineering applications.

## 2. Seismic damage prediction methods for buildings on seismic ruptures

The degree of damage or seismic damage to buildings on sites adjacent to strong earthquake surface rupture zones is an analysis process of the damage response of different types of buildings under various influencing factors of surface faults. Simply put, it is the seismic damage analysis of buildings caused by surface faults. This problem can be attributed to the multivariate statistical prediction problem. The so-called diversity refers to two aspects. One is that the rupture parameters of surface faults, such as fault type, fault dip angle, site conditions, and other variables, all affect the degree of seismic damage to buildings; On the other hand, in the case where multiple variables of surface faults are the same, the structural type, foundation type, and number of floors of a building can also affect the degree of damage to the building. For the influencing factors of buildings in the latter, combined with regulations and previous research, the seismic damage index can be used as an important indicator, because the seismic damage index is a numerical value between 0 and 1. By quantitatively evaluating the specific degree of damage caused by earthquakes to buildings of different structural types, it provides scientific basis for earthquake relief and post disaster reconstruction.

**Fig 2:**
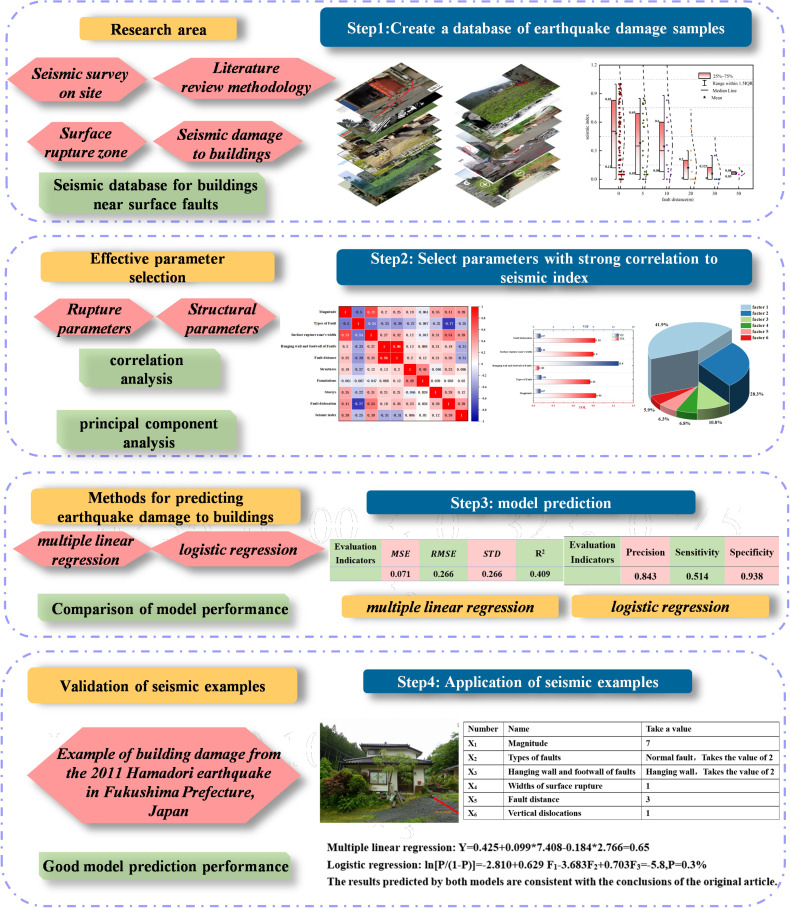
Article context and process.

Therefore, based on considering multiple influencing factors, this article constructs a reasonable sample database and provides a method for predicting building damage on earthquake prone faults, quantitatively analyzing the relationship between surface rupture parameters and building damage indices. The specific process is shown in Fig 2:

(1) Establish a database. The collection and organization of surface rupture parameters and building damage parameters is the main part of the database, analyzing and extracting factors such as earthquake information, site damage information, fracture information, and building damage information to establish a database of building damage near earthquake surface rupture zones.
(2) Select valid parameters. By using correlation analysis and principal component analysis, the factors in the building damage database are diagnosed and effective parameters are obtained for inclusion in the regression model.
(3) Establish a regression model. Perform linear regression and logistic regression analysis on the dependent variable and effective parameters to predict building damage near surface faults under the influence of multiple factors.
(4) Introduce earthquake damage cases. Using earthquake damage examples, test the predictive performance of two models.

### 2.1. Database creation

For studying the risk of surface rupture and building structural damage in engineering sites, historical data on the degree of building damage on strong earthquake faults is very valuable. Therefore, establishing a reasonable database and sharing it can help promote research on the damage mechanism and seismic measures of building structures near the fault of the earthquake.

#### (i) Data source.

The data sources of strong earthquake surface rupture are scattered and span a long time, and the identification of the building damage index requires certain professional technical knowledge.

This article collects a large amount of seismic information on faults and engineering structures along the surface rupture line at home and abroad. Specifically, these data come from major earthquakes at home and abroad. The sources of surface rupture and building damage data in the damage database are mainly journal articles, geotechnical investigation reports, and local log records. The data sources are relatively scattered, and there may be discrepancies between data from different sources, which requires the necessary screening of the data during statistical analysis. Partial data can be found in Appendix 1.

#### (ii) Database content.

The database of building damage near the strong earthquake surface rupture zone established includes: (1) earthquake information: earthquake area, earthquake time, earthquake name, magnitude, and focal depth;(2) site damage information: site category, overburden thickness, site damage situation;(3) rupture zone information: types of fault, surface rupture zone’s width, vertical dislocation;.(4)building damage information: building name, total number of floors, types of structure, types of foundation, Hanging wall and footwall of faults, fault distance, seismic damage index, and building damage information.

The influencing factor, as a feature input of the regression model, is almost crucial in terms of reliability. The magnitude, focal depth, types of fault, and width of the rupture zone play an important role in the seismic damage index. The types of structural, types of foundation, number of floors, vertical displacement, horizontal displacement, fault distance, and location of a building directly affect the magnitude of the seismic damage index. Therefore, after comprehensive consideration, the above influencing factors will be used as the parameter sources for constructing the database.

#### (iii) Database format.

In the process of data collection and statistics, the database consists of Excel spreadsheets and PDF files. Excel spreadsheets have simple search and statistical functions; Detailed information such as photos of surface rupture zones and building damage can be found in PDF files.

### 2.2. Prediction method

Linear regression, as an important method in the field of statistics, is mainly applied to describe the relationship between independent and dependent variables. However, due to the precise data required for its application and the need for a linear distribution between the independent and dependent variables, using traditional linear regression methods in many practical problems can encounter many difficulties, resulting in inaccurate prediction results. Although logistic regression is essentially a generalized linear model similar to linear regression, it is mainly used for data classification rather than dealing with regression problems. Generally speaking, logistic regression divides precise data into multiple categories based on certain classification criteria for prediction, and each category has a specific parameter vector. But as the number of categories increases, the interpretability of the parameter vector decreases, while the uncertainty increases, thereby reducing the predictive performance of logistic regression. Therefore, this article will compare traditional machine learning algorithms - linear regression and logistic regression to explore the seismic damage of buildings near surface faults under the influence of multiple factors [37].

This article uses IBM SPSS Statistics 27.0 simulation software for data entry, organization, review, and related statistical analysis to evaluate multiple linear regression models and binary logic models.

#### 2.2.1. Correlation analysis.

**Fig 3:**
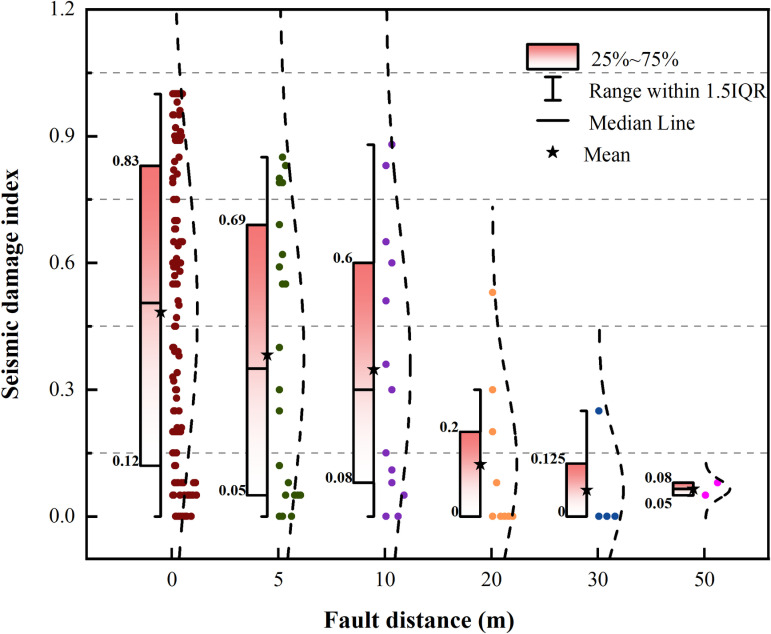
Trend chart of seismic damage index with increasing fault distance.

Previous studies have only analyzed the relationship between the building damage index and distance from the surface rupture zone. This article combines the established database of building damage near typical earthquake surface rupture zones and also analyzes the trend of damage index variation with fault distance, as shown in Fig 3. This figure indicates that the seismic damage index is not only determined by the distance between faults but also influenced by many other rupture parameters. Factors such as the width of the rupture zone, the type of fault, as well as the structural and foundation types of buildings, all closely affect the seismic damage index. Therefore, it is necessary to comprehensively examine the quantitative relationship of seismic damage index under the influence of multiple factors.

This article preliminarily suggests that there are 10 independent variables X closely related to the building damage index (magnitude, fault type, rupture zone width, hanging wall and footwall of fault, fault distance, structural type, foundation type, number of floors, vertical displacement, horizontal displacement), and only 1 dependent variable Y (damage index). The definition and meaning of categorical variables are shown in Table 1.

**Table 1:** Classification variable names and meanings.

Variable	Meaning
Fault type	1 = Reverse fault；2 = Normal fault；3 = Strike-slip fault.
Hanging wall and footwall of fault	1 = Cross trace line；2 = Hanging wall；3 = Footwall.
Structural type	1 = Steel；2 = Frame；3 = Reinforced concrete；4 = Timber；5 = Brick concrete；6 = Brick and wood.
Foundation type	1 = Box；2 = Raft；3 = Pile；4 = Independent；5 = Strip.

**Fig 4:**
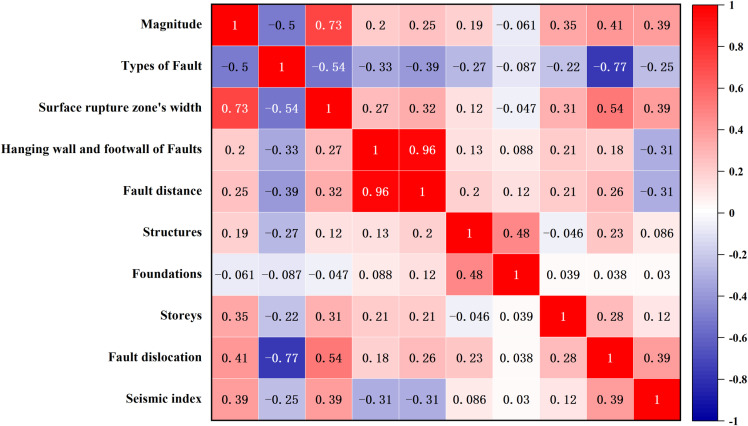
Correlation heatmap between different influencing factors and seismic damage index.

**Fig 5:**
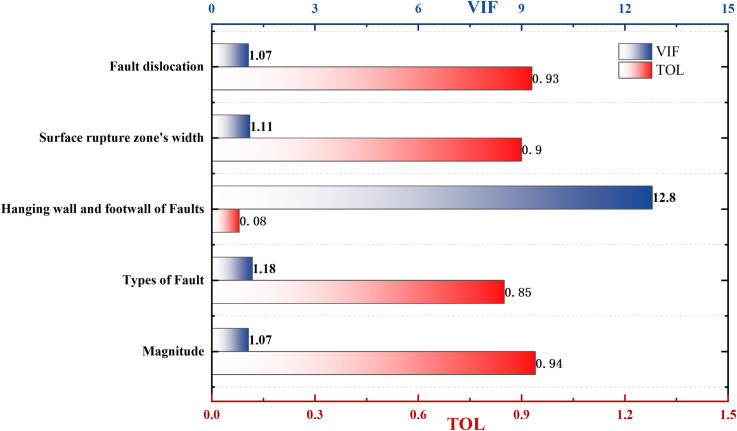
Correlation analysis between fault distance and other factors.

To clarify the degree of correlation between the seismic damage index and these surface fault factors, this article first conducted Spearman-level correlation analysis and drew a multi-factor correlation heatmap, as shown in Fig 4. The seismic damage index is significantly correlated with six factors, including magnitude, fault type, hanging wall and footwall of faults, rupture zone width, fault distance, and vertical displacement. In addition, the correlation coefficient between the fault distance and the position of the hanging wall and footwall of faults is 0.96, and the correlation coefficient between the magnitude and rupture zone width is 0.73, both of which show a highly significant correlation. Considering only the correlation strength between influencing factors is not convincing. Based on this, a multicollinearity analysis was conducted on the influencing factors, as shown in Fig 5. Tolerance (abbreviated as TOL) and Variance inflation factor (abbreviated as VIF)values are measures of the severity of multicollinearity among multiple variables. From the perspective of fault distance, the position of buildings is closely related to it, with TOL and VIF of 0.08 and 12.8, respectively, exceeding their respective threshold values, indicating severe collinearity.

The overall stability of building structures is positively correlated with their ability to withstand large earthquakes, but this does not mean that they can more effectively cope with surface fractures. When underground bedrock faults move and penetrate to the surface, a surface main fault zone is formed, accompanied by surface coseismic dislocations along the line, causing buildings located on the fault zone to tilt or even collapse due to surface displacement. This study focuses on earthquake damage samples of buildings adjacent to surface fault areas. Therefore, the surface rupture effect caused by strong earthquakes is significant, making the advantages of different structural types less apparent in this situation. Similarly, the foundations of buildings exhibit significant differences in resisting surface fault ruptures. As a connecting bridge between buildings and foundations, the foundation carries the entire weight of the building and transfers it to the foundation. Strong earthquake-induced surface rupture may cause deformation or damage to the foundation soil, thereby affecting the stability of the foundation. However, whether the foundation can effectively resist surface rupture to protect the superstructure also depends on the severity and scope of the rupture. In extreme cases of surface rupture, even high-quality foundation designs may be difficult to fully withstand. Given that this article focuses on seismic damage samples in areas adjacent to surface faults, the surface rupture effect of strong earthquakes is significant, and therefore the advantages of different foundation types are no longer prominent. There is no direct correlation between the degree of damage to a building and its number of floors. Evaluating the seismic damage index of a building requires comprehensive consideration of multiple factors such as its structural type, material properties, foundation conditions, seismic design standards, and seismic rupture characteristics. Therefore, the seismic damage index of a building does not solely depend on its structural type, foundation type, or number of floors. There is a close correlation between the seismic damage index and the rupture parameters of earthquakes, such as magnitude, fault type, width of surface rupture zone, and fault distance. At the same time, the relative position relationship between buildings and faults also has an important impact on the degree of seismic damage. These rupture parameters directly reveal the extent and scope of damage caused by earthquakes to the surface and buildings above it.

**Fig 6:**
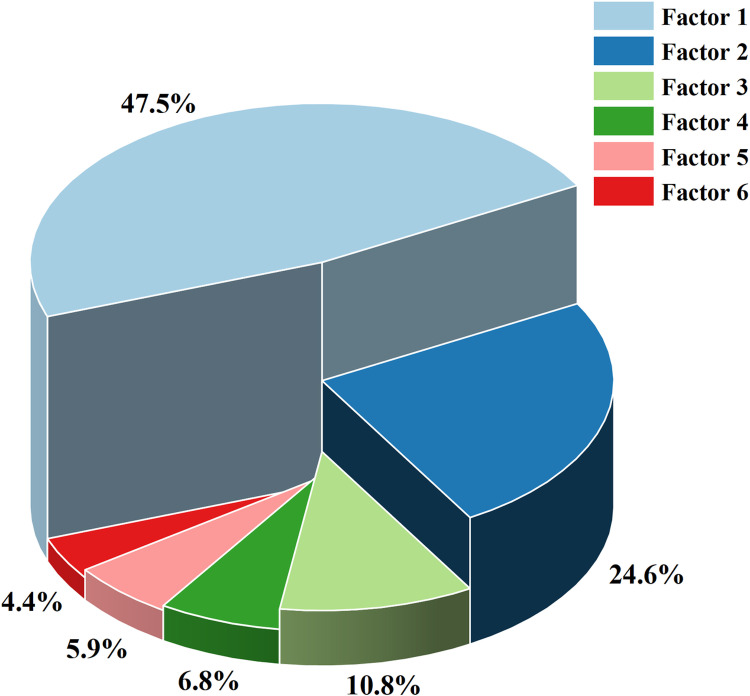
Proportion chart of principal component factors.

This article decides to use Principal Component Analysis (abbreviated as PCA) to solve the problem of collinearity. PCA identifies the main components in the data (which are linear combinations of the original variables), maps the original variables to a low dimensional space, and preserves the main feature information of the data as much as possible, thereby reducing the number of independent variables. From Fig 6, it can be seen that the three-dimensional pie chart arranges the principal component factors in descending order of percentage, with the first three factors accounting for 47.5%, 24.6%, and 10.8% respectively, totalling 83% of the total impact effect, denoted as F_1_, F_2_, F_3_, as a new feature variable for subsequent multiple linear regression. Table 2 shows the component score coefficient matrix, which details the score coefficients for each original variable corresponding to the three principal components. These coefficients quantify the closeness of the linear relationship between the original variables and the principal components, and derive the specific expression of the principal component factors as follows:

**Table 2:** Matrix of component score coefficients.

Factors	Ingredients		
	1	2	3
**Magnitude**	0.774	-0.160	0.452
**Hanging wall and footwall of faults**	0.337	0.845	0.019
**Types of fault**	-0.829	0.187	0.337
**The width of surface rupture**	0.790	-0.160	0.376
**Fault distance**	0.468	0.762	-0.097
**Vertical dislocation**	0.780	-0.304	-0.421


$$F_{1}=0.774X_{1}+0.337X_{2}-0.829X_{3}+0.790X_{4}+0.468X_{5}+0.780X_{6}$$

$$F_{2}=-0.160X_{1}+0.845X_{2}+0.187X_{3}-0.160X_{4}+0.762X_{5}-0.304X_{6}$$

$$F_{3}=0.452X_{1}+0.019X_{2}+0.337X_{3}+0.376X_{4}-0.097X_{5}-0.421X_{6}$$

#### 2.2.2. Multiple linear regression model.

The multiple linear regression model is used to study the linear relationship between a dependent variable and multiple independent variables. Its basic principle is to describe the relationship between the dependent variable and the independent variable by establishing a linear equation and to estimate the parameters in the equation using sample data. This linear equation can be expressed as:

$Y=\beta_{0}+\beta_{1}X_{1}+\beta_{2}X_{2}+\ldots +\beta_{k}X_{k}+\varepsilon$

For the multiple linear regression model in this article, the independent variables are the three characteristic variables after principal component analysis(F1, F2, F3), and the dependent variable is the seismic damage index of the building. The linear regression algorithm has multiple judgment indicators for model evaluation, and this article will focus on using mean square error and coefficient of determination to evaluate the accuracy of the final linear model. Mean square error is a measure that reflects the degree of difference between an estimated value and the estimated value, while the coefficient of determination is a measure used to reflect how much percentage of the fluctuation of the dependent variable can be described by the fluctuation of the independent variable. Generally speaking, the smaller the mean square error (MSE) value, the smaller the error between the predicted and actual values output by the model, and the higher the accuracy. On the contrary, the higher the decision coefficient R-value, the better the model.

#### 2.2.3. Binary logistic regression model.

The binary logistic regression model is an important method used in statistics to analyze the relationship between binary dependent variables and independent variables. In binary logistic regression models, the dependent variable can only take two values, usually 1 and 0, representing two possible outcomes (such as yes or no, success or failure, etc.). The independent variable can be quantitative or categorical data used to explain or predict the value of the dependent variable. The model expression is:


$$logit(P)=ln[P/\;(1-P)]=\beta_{0}+\beta_{1}X_{1}+\beta_{2}X_{2}+\ldots +\beta_{k}X_{k}$$

The dependent variables in this article have values of 1 and 0, representing two possible outcomes (whether the building collapses or not). Among them, if the building collapses, take 1, and if the building does not collapse, take 0. Assuming P is the probability of collapse occurring, then 1-P is the probability of no collapse occurring. In evaluating the logistic regression model, three evaluation indicators, accuracy, sensitivity, and specificity, will be used to assess the final accuracy of the binary logistic regression model.

## 3. Results and discussion

### 3.1. Multiple linear regression results

All sample data were used to construct a multiple linear regression model, and multiple linear regression analysis was conducted between the three characteristic variables (F_1_, F_2_, F_3_) and the seismic damage index (Y). The regression equation is Y = 0.425 + 0.099 F_1_-0.184F_2_, as shown in Table 3. The P-values of constant coefficient, F_1_, and F_2_ are all below 0.001, accepting the null hypothesis that they have a positive impact on the seismic damage index; The P-value of F_3_ is 0.116, which is greater than 0.05, so the feature variable F_3_ is not included in the model. According to Table 4, R^2^ reveals that the independent variable can explain the differences in the dependent variable to a degree of 35%. The significance test P value of the model is less than 0.001, indicating that the model has a good fit. Mean square error, root mean square error, mean absolute error, and standard deviation also provide dual guarantees for the reliability of the model, as shown in Table 5.

**Table 3:** Multiple linear regression coefficients.

Modelling	Non-standardized coefficient		Standardized coefficient		
	B	Standard error	Beta	t	Sig
C	0.425	0.022		19.449	<0.001
F_1_	0.099	0.022	0.284	4.535	<0.001
F_2_	-0.184	0.022	-0.525	-8.388	<0.001
F_3_	0.035	0.022	0.099	1.581	0.116

**Table 4:** Model Summary.

R	R^2^	Adjusted R^2^	Error in standard estimation	Change in P-value
0.605	0.366	0.354	0.28	<0.001

**Table 5:** Evaluation indicators.

MSE	RMSE	STD	R^2^
0.079	0.28	0.28	0.354

### 3.2. Logistic regression results

All sample data were used to construct a binary logistic regression model, with three feature variables (F_1_, F_2_, F_3_) and a binary categorical variable (occurrence of casualties and non-occurrence of casualties). The regression equation is ln [P/(1-P)]=-2.810 + 0.629 F_1_-3.683F_2_+0.703F_3_, as shown in Table 6. The P-values of constant coefficients, F_1_, F_2_, and F_3_ are all below 0.001, supporting the null hypothesis. The significance of the Hosmer Lemeshow test is greater than 0.05, indicating the validity of this model, as shown in Table 7. The accuracy, sensitivity, and specificity of the binary logistic regression model obtained from the confusion matrix also ensure the accuracy of the model, as shown in Tables 8 and 9.

**Table 6:** Binary logistic regression coefficients.

	B	Standard error	Wald	Sig	Exp(B)
C	-2.810	0.269	5.453	0.020	1.875
F_1_	0.629	0.767	23.087	<0.001	0.025
F_2_	-3.683	0.284	6.109	0.013	2.019
F_3_	0.703	0.463	36.820	<0.001	0.060

**Table 7:** Hosmer Lemeshau test.

Chi-square	Freedom	Sig
11.166	8	0.192

**Table 8:** Confusion matrix.

True value	Predictive value	
	0	1
**0**	**122**	**7**
**1**	**19**	**18**

**Table 9:** Evaluation indicators.

Precision	Sensitivity	Specificity
0.843	0.486	0.946

### 3.3. Application of earthquake damage examples

To test the predictive performance of the two models, this paper extracted a building damage example (P17) from the 2011 Hamato earthquake in Fukushima Prefecture, Japan [38]. It is a relatively ancient wooden house, with a surface fault at the right end and a left inclination of about 5 degrees. The original text sets its degree of damage as D4. The magnitude of this earthquake is 7, and the building is located on the hanging wall of a normal fault. The width of the surface rupture zone is about 1 meter, and the distance between the building and the surface rupture zone is about 3 meters. The vertical displacement of the surface fault is about 1 meter, as shown in Table 10.

**Table 10:** Variable name and value.

Variable number	Variable	Value
X1	Magnitude	7
X2	Hanging wall and footwall of faults	Normal fault = 2
X3	Types of fault	Hanging wall = 2
X4	The width of surface rupture	1
X5	Fault distance	2
X6	Vertical dislocation	1

Three feature variables are obtained by converting six original variables into coefficient matrices:


$$F_{1}=0.774*7+0.337*2-0.829*2+0.790*1+0.468*3+0.780*1=7.408$$

$$F_{2}=-0.160*7+0.845*2+0.187*2-0.160*1+0.762*3-0.304*1=2.766$$

$$F_{3}=0.452*7+0.019*2+0.337*2+0.376*1-0.097*3-0.421*1=3.54$$

Linear regression model prediction results: Y = 0.425 + 0.099*7.408-0.184*2.766 = 0.65, the predicted seismic damage index value is 0.65, indicating that the building is in a state of moderate damage, which corresponds to the conclusion of the original text. Binary logistic regression model prediction results: ln[P/ (1-P)]=-2.810 + 0.629*7.408-3.683*2.766 + 0.703*3.54 = -5.8, the calculated P = 0.3% indicates that the probability of this building collapsing is extremely low, about 0.3%. Obviously, the wooden building in the original text did not collapse, which corresponds to the conclusion of this article.

The results of the practical application have confirmed the good predictive performance of the two models, providing more refined considerations and targeted designs for seismic fortification of near fault buildings, achieving an organic combination of scientific research innovation and engineering applications.

### 3.4. Analysis and discussion

From the above results, it can be found that the multiple linear regression model can explain the dependent variable to a degree of 35%. After classification prediction using the logistic regression model, the accuracy of the model is about 85%, indicating that logistic regression has significantly better performance in predicting building damage datasets. In the building damage sample dataset used in this article, independent variables with obvious discrete characteristics such as fault types are included. Therefore, when using a linear regression model to solve the regression problem of this dataset, the weak linear relationship between the independent variables and the dependent variable leads to a lower R^2^ between them. For logistic regression, its model does not require a strong linear relationship between the independent and dependent variables and will discretize continuous dependent variable values into different category values (0 and 1 in this article) for classification prediction. Therefore, when using machine learning to solve problems, the selection of methods is largely related to the features of the data. Based on the discrete characteristics of the building damage dataset, this paper will adopt more methods to predict the building damage index in future research work, providing relevant methods for earthquake damage prediction for subsequent studies by relevant scholars.

## 4. Conclusions

The degree of seismic damage to building structures near the surface rupture zone is usually higher than that far away from the area, and this degree of seismic damage is highly correlated with the parameters of fault rupture. Although countries have proposed specific values for avoidance distance, there is a lack of sufficient and reasonable explanation and verification, which may constrain urban planning and development. Therefore, a more detailed study is needed on the relationship between rupture zones and buildings. The research and development process can be divided into two stages: the first stage focuses on the analysis of the degree of surface rupture in free sites; In the second stage, surface rupture and the resulting structural damage will gradually become the research core.

Theoretical analysis shows that the investigation and analysis of building damage is the most direct and effective method to reveal its mechanism, but the statistical and analytical data of building damage samples near surface faults are clearly insufficient. Therefore, based on the influencing factors of building damage near surface faults, this article predicts the structural damage caused by the surface rupture effect of active faults. This article collects data on the seismic damage characteristics of buildings near surface fault zones through on-site investigation and literature review methods, establishes a seismic damage database for buildings near strong earthquake surface fault zones, and quantitatively analyzes the seismic damage of buildings using multiple linear and binary logistic regression models.

(1) The on-site seismic investigation shows that the damage to buildings near the surface rupture zone is closely related to various factors. Therefore, this article has refined and established a seismic damage database, which covers detailed content such as earthquake information, site damage, fault zone characteristics, and building damage. Due to the scattered data sources and the need for professional knowledge identification, strict screening was conducted during the statistical analysis. The data is sourced from journal literature on major earthquakes both domestically and internationally, geotechnical investigation reports, and local logs. It is presented in Excel spreadsheets and PDF files for easy search, statistics, and detailed queries, including detailed information such as photos. This database provides valuable resources for in-depth analysis of building damage near surface rupture zones.
(2) To reasonably predict the seismic damage index of buildings near faults, this paper identifies the influence weights of various factors, provides a prediction model for building seismic damage under the influence of multiple factors, and conducts quantitative analysis.

The expression of the multiple linear regression model is Y = 0.425 + 0.099F_1_-0.184F_2_, with a mean square error of 0.079 and a standard deviation of 0.28; The expression of the binary logistic regression model is In [P/(1-P) 1 = -2.810 + 0.629F_1_-3.683F_2_+0.703F_3_, with an accuracy of 0.85. From the evaluation indicators, it is preliminarily believed that the two models have a good fit.

(3) To verify the accuracy of the predictive performance of the two models, this paper introduces a typical case of near fault building damage. After verification, the linear regression model predicts a seismic damage index of 0.65 for buildings, and the binary logistic regression model predicts a probability of 0.3% for building collapse, both of which are consistent with the actual seismic damage situation of the building in the original text, indicating that the two prediction models in this paper have good performance.

## Supporting information

S1 Part of the original data. (DOCX)
