# Supplementary material for: Predictive analysis of seismic damage to buildings near-surface faults under the influence of multiple factors
Source: PLoS One. 2025 May 7;20(5):e0320930. doi: 10.1371/journal.pone.0320930 (PMC12057976; doi:10.1371/journal.pone.0320930)
Supplement: S1 — (DOCX) [file pone.0320930.s001.docx]

**Supporting Information**

S1. Part of the original data.

|  | **Name** | **Structure**  **types** | **Foundation types** | **Magnitude** | **Position** | **Fault types** | **Surface fracture**  **zone** | **Distance to surface rupture zone** | **Vertical displacement** | **Horizontal displacement** | **Seismic damage index** | **Source** |
| --- | --- | --- | --- | --- | --- | --- | --- | --- | --- | --- | --- | --- |
| **1** | **4-storey building in Midtown Park, Fungam** | **3** | **2** | **7.6** | **1** | **1** | **12** | **0** | **4** | **0** | **0.8** | **[1][2]** |
| **2** | **Three-storey RC residence** | **3** | **5** | **7.6** | **1** | **1** | **5** | **0** | **3** | **0** | **0.82** | **[3]** |
| **3** | **4-storey building in Fungam City** | **3** | **5** | **7.6** | **2** | **1** | **20** | **0** | **0.63** | **0** | **0.75** | **[1]** |
| **4** | **Yellow buildings in the Hassa area** | **1** | **5** | **7.8** | **1** | **3** | **3** | **0** | **0** | **2** | **0.55** | **[5]** |
| **5** | **A house in the Hassa area** | **3** | **5** | **7.8** | **1** | **3** | **15** | **0** | **0** | **3** | **0.9** | **[6]** |
| **6** | **Building next to Yingxiu Highway** | **2** | **4** | **8** | **1** | **1** | **20** | **0** | **2** | **0** | **0.65** | **[15]** |
| **7** | **Beichuan Shaba Village B** | **5** | **5** | **8** | **2** | **2** | **36** | **8** | **1.5** | **0** | **0.51** | **[16]** |
| **8** | **Tongji Township Shuangyang villagers 2-storey building** | **5** | **5** | **8** | **2** | **1** | **12.9** | **30** | **0** | **0** | **0.25** | **[7]** |
| **9** | **Beichuan County Party Committee Building** | **5** | **5** | **8** | **1** | **1** | **10** | **0** | **3** | **2** | **1** | **[8]** |
| **10** | **Wooden house** | **4** | **2** | **7.1** | **1** | **3** | **2** | **0** | **0** | **0.5** | **0.68** | **[9]** |
| **11** | **Wooden farm shed** | **4** | **3** | **7.1** | **1** | **3** | **15** | **0** | **0** | **2.7** | **0.33** | **[10]** |
| **12** | **Light industrial buildings** | **1** | **2** | **7.1** | **1** | **3** | **40** | **0** | **0.1** | **1.7** | **0.15** | **[10]** |
| **13** | **Building 1** | **3** | **1** | **7.4** | **2** | **2** | **15** | **2** | **2.3** | **1.1** | **0.25** | **[11]** |
| **14** | **Findikli village building 2** | **4** | **5** | **7.4** | **1** | **3** | **2** | **0** | **0.8** | **0.5** | **0.7** | **[12]** |
| **15** | **The Mosque** | **3** | **4** | **7.4** | **1** | **2** | **6** | **0** | **1.3** | **0** | **0.89** | **[11]** |
| **16** | **Koran School** | **5** | **5** | **7.4** | **1** | **3** | **1** | **0** | **0** | **1** | **0.25** | **[4]** |
| **17** | **Residence in Arifive** | **3** | **2** | **7.4** | **1** | **3** | **1** | **0** | **0** | **0.1** | **0.08** | **[4]** |
| **18** | **The "Attaturk" basketball court** | **3** | **3** | **7.4** | **1** | **2** | **1** | **0** | **1.5** | **0.9** | **0.68** | **[4]** |
| **19** | **Primary School in Kullar** | **3** | **3** | **7.4** | **1** | **2** | **3** | **0** | **0.5** | **2** | **0.91** | **[4]** |
| **20** | **Wufeng Apartment Building** | **2** | **4** | **7.6** | **1** | **1** | **20** | **0** | **2** | **0** | **0.6** | **[4]** |
| **21** | **Warehouse** | **3** | **5** | **7.6** | **2** | **1** | **15** | **3** | **2** | **0** | **0.8** | **[14]** |
| **22** | **Yesilyurt building** | **3** | **5** | **7.8** | **1** | **3** | **2** | **0** | **0** | **2.8** | **0.9** | **[6]** |
| **23** | **Northwest Teaching Building of Bailu Middle School** | **5** | **5** | **8** | **2** | **1** | **18** | **6** | **0** | **0** | **0** | **[15]** |
| **24** | **Hongxiangkou Bajiao Temple Rest Home** | **5** | **5** | **8** | **2** | **1** | **22** | **20** | **0** | **0** | **0.08** | **[15]** |
| **25** | **Building 2 by the River in Yingxiu Town** | **5** | **5** | **8** | **1** | **1** | **40** | **0** | **3** | **0** | **1** | **[16]** |
| **26** | **Residential buildings in Xiaoyudong Town** | **5** | **5** | **8** | **1** | **1** | **20** | **0** | **1** | **2** | **1** | **[16]** |
| **27** | **2-story building and 1 attic** | **3** | **1** | **7.4** | **3** | **2** | **1.5** | **1** | **0** | **0** | **0.05** | **[14]** |
| **28** | **Manor House** | **4** | **3** | **7.8** | **2** | **1** | **10** | **5** | **0.5** | **0** | **0.59** | **[13]** |
| **29** | **Paradise Cottage** | **4** | **3** | **7.8** | **2** | **1** | **12** | **1** | **0** | **0.1** | **0.83** | **[13]** |
| **30** | **One story house on the west side of the central hill** | **5** | **5** | **7.8** | **1** | **1** | **5** | **0** | **1** | **0** | **0.9** | **[17]** |
| **31** | **Wooden buildings** | **4** | **5** | **7** | **1** | **2** | **2** | **0** | **0.3** | **0** | **0.08** | **[3]** |
| **32** | **Contra Costa College** | **3** | **4** | **6.8** | **1** | **3** | **0.5** | **0** | **0** | **0.1** | **0.05** | **[18]** |
| **33** | **Building 1** | **5** | **5** | **6.3** | **1** | **2** | **1** | **0** | **0.06** | **0** | **0.5** | **[19]** |
| **34** | **Batdorf building** | **4** | **2** | **7.3** | **1** | **3** | **2** | **0** | **0** | **0.2** | **0.2** | **[20]** |
| **35** | **Building 2** | **5** | **5** | **6.7** | **2** | **1** | **4** | **10** | **0** | **0** | **0.3** | **[21]** |
| **36** | **Site C1** | **5** | **5** | **6.2** | **1** | **3** | **1** | **0** | **0.4** | **0.77** | **0.45** | **[22]** |

Structure types: 1-Steel structure; 2-Frame structure; 3-Reinforced concrete structure; 4-Timber structure; 5-Brick concrete structure; 6-Brick and wood structure.

Foundation types: 1-Box foundation; 2-raft foundation; 3-Pile foundation; 4-Independent foundation; 5-Strip foundation.

Position: 1-cross trace line; 2-Hanging wall; 3-Footwall.

Fault types: 1-Reverse fault; 2-Normal fault; 3-Strike-slip fault.

**References**

1. Faccioli E, Anastasopoulos I, Gazetas G, Callerio A, Paolucci R. Fault rupture foundation interaction: selected case histories. Bull Earthquake Eng. 2008;6:557-583.
2. Su N, Lin TD, Chai HW. Damage to structures and buildings from the Chi-Chi (Taiwan) earthquake. Proceedings of the Institution of Civil Engineers-Structures and Building. 2002;152(1):51-56.
3. Hisada Y, Tanaka S, Kaneda J. Investigation of Building Damage near Surface Fault Rupture of the 2016 Kumamoto Earthquake and Countermeasures for Active Faults. Journal of Japan Association for Earthquake Engineering. 2020;20(2): 90-132.
4. Redmond L. Survey of surface fault rupture and structure interaction. D, Cal Poly. California, 2012.
5. Feng J, Yan JY, Zhao XX. Preliminary Investigation on the Surface Ruptures of the Turkey M7.8 Earthquake on February 6, 2023. Beijing Da Xue Xue Bao Zi Ran Ke Xue Bao. 2023;59(6):945-950.
6. Mavroulis S, Argyropoulos I, Vassilakis E, Carydis P. Earthquake Environmental Effects and Building Properties Controlling Damage Caused by the 6 February 2023 Earthquakes in East Anatolia. Geosciences. 2023;13(10):303.
7. Zhou Q, Xu XW, Yu GH, Chen XC, H HL, Yin GM. Investigation on widths of surface rupture zones of the M8.0 Wenchuan earthquake, Sichuan Province, China. Seismology and Geology. 2008;30(3):778-788.
8. Guo TT, Yu GH, Xu XW. Characteristics of Wenchuan Earthquake Disasters and Discussion on Causes of Buildings Damage. Earthquake Resistant Engineering and Retrofitting. 2010.
9. Quigley M, Dissen RV, Villamor P, Litchfield N, Barrell D, Furlong K, et al. Surface rupture of the Greendale fault during the Darfield (Canterbury) earthquake, New Zealand: initial findings. Bulletin of the New Zealand Society for Earthquake Engineering. 2010;43(4):236-242.
10. Dissen RV, Barrell D, Litchfield N, Villamor P, Quigley M, King A, et al. Surface rupture displacement on the Greendale Fault during the M w 7.1 Darfield (Canterbury) earthquake, New Zealand, and its impact on man-made structures. Proceedings of the Ninth Pacific Conference on Earthquake Engineering. 2011.
11. Anastasopoulos I, Gazetas G. Foundation–structure systems over a rupturing normal fault: Part I. Observations after the Kocaeli 1999 earthquake. Bull Earthquake Eng. 2007;5:253-275.
12. Ulusay R, Aydan O, Hamada M. The behaviour of structures built on active fault zones: examples from the recent earthquakes of Turkey. Structural Eng. 2002;19(2):149-167.
13. Dissen RJV, Stahl T, King A, Pettinga JR, Fenton C, Little TA, et al. Impacts of surface fault rupture on residential structures during the 2016 Mw 7.8 Kaikōura earthquake, New Zealand. Bulletin of the New Zealand Society for Earthquake Engineering. 2019;52(1):1-22.
14. Bray JD.Developing mitigation measures for the hazards associated with earthquake surface fault rupture[J].Workshop on Seismic Fault, 1960.
15. Guo TT. A Study on Wenchuan Earthquake Disasters and the Safety Distance from Active Faults. D, Institute of Geology, China Earthquake Administrator. Beijing, 2013.
16. Zhang JY. Active Fault Setback Research on Engineering Sites. D, Institute of Engineering Mechanics, China Earthquake Administration. Heilongjiang, 2015.
17. Mahmood K, Zamin B, Iqbal S, Rehman ZU, Afzal S, Safdar M, et al.Local site effect on seismic hazard of the relocated new Balakot town.Soil Dynamics and Earthquake Engineering. 2022:107451.
18. Stoffer PW. Where's the Hayward Fault? A Green Guide to the Fault. US Geological Survey. 2008.
19. Boncio P, Galli P, Naso G, Pizzi A. Zoning surface rupture hazard along normal faults: Insight from the 2009 Mw 6.3 L'Aquila, central Italy, earthquake and other global earthquakes. Bulletin of the Seismological Society of America. 2012;102(3):918-935.
20. Murbach D, Rockwell TK, Bray JD.The relationship of foundation deformation to surface and near-surface faulting resulting from the 1992 Landers earthquake. Drug Delivery. 1996;22(32):1-6.
21. ISHIMURA D, OKADA S, NIWA Y, TODA S. The surface rupture of the 22 November 2014 Nagano-ken-hokubu earthquake (Mw 6.2), along the Kamishiro fault. Japan, Active Fault Research. 2015;43:95-108.
22. Huang SY, Yen JY, Wu BL, Yen IC, Chuang RY. Investigating the Milun Fault: The coseismic surface rupture zone of the 2018/02/06 M_L_ 6.2 Hualien earthquake, Taiwan. Terrestrial Atmospheric and Oceanic Sciences. 2019;3.
